# Supplementary material for: A Potential Alternative Orodispersible Formulation to Prednisolone Sodium Phosphate Orally Disintegrating Tablets
Source: Pharmaceutics. 2021 Jan 19;13(1):120. doi: 10.3390/pharmaceutics13010120 (PMC7832848; doi:10.3390/pharmaceutics13010120)
Supplement: Supplementary file 1 [file pharmaceutics-13-00120-s001.pdf]

pharmaceutics

Supplementary Materials: A Potential Alternative Orodispersible Formulation to Prednisolone Sodium Phosphate Orally Disintegrating Tablets

Essam A. Tawfik, Mariagiovanna Scarpa, Hend E. Abdelhakim, Haitham A. Bukhary, Duncan Q. M. Craig, Susan A. Barker and Mine Orlu

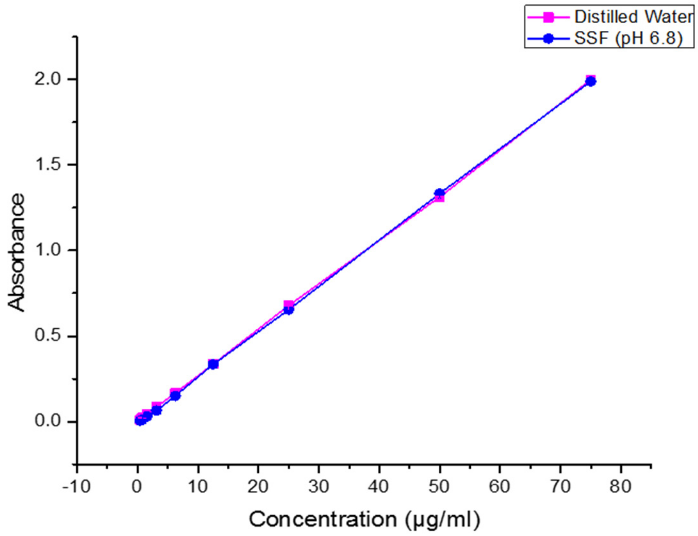

Figure S1. PSP calibration curves in distilled water and SSF (pH 6.8) for measuring PSP drug loading and in vitro release, respectively. Both curves showed excellent linearity at a concentration range of 75 to 0.39 µg/mL, with the $r^2 > 0.999$.
